# The Prevalence of Plasmid-mediated Quinolone Resistance Genes in *Escherichia coli* Isolated from Hospital Wastewater Sources in Tehran, Iran

**Published:** 2017-09

**Authors:** Reza RANJBAR, Omid FARAHANI

**Affiliations:** 1. Molecular Biology Research Center, Baqiyatallah University of Medical Sciences, Tehran, Iran; 2. Dept. of Microbiology, Islamic Azad University, Varamin-Pishva Branch, Tehran, Iran

**Keywords:** *Escherichia coli*, Quinolone, Hospital wastewater, Iran

## Abstract

**Background:**

Considering the importance of hospital wastewaters as potential reservoirs for the dissemination of bacterial pathogens such as *Escherichia coli* and antibiotic resistance genes in the environment, the need for such information becomes imperative.

**Methods:**

*E. coli* strains were isolated from hospital wastewater sources in Tehran, Iran, over a 24-month sampling period (Jun 2014– Jun 2016) and identified using standard bacteriological methods. Quinolone resistance among the strains was determined using Kirby-Bauer method and the frequency of plasmid-mediated quinolone resistance genes (*qnrA*, *qnrB*, *qnrS*) was investigated by PCR.

**Results:**

In total, 80 *E. coli* strains were isolated during the study period, of which 51 (63.8%) isolates were resistant to tested antibiotics. Of note, 13 isolates were resistant to all the antibiotics employed. The highest rates of antibiotic resistance were obtained for nalidixic acid (60%), followed by norfloxacin (30%), and ciprofloxacin (25%). Of the 51 quinolone-resistant strains, 24 (47.1%) isolates harbored *qnr* genes. None of the isolates harboured the *qnrA* gene, while 11 (45.8%) and 7 (29.2%) isolates contained *qnrB* and *qnrS*, respectively.

**Conclusion:**

Our findings showed high rates of quinolone resistance (63.8%) and *qnr* genes, underlining the importance of hospital wastewaters as reservoirs for dissemination of potentially pathogenic *E. coli* and horizontal gene transfer between other waterborne bacterial species. Other possible mechanisms of resistance should also be investigated for better characterization of quinolone-resistant *E. coli* isolates.

## Introduction

Hospitals wastewater is dangerous for health and environment because it likely contains many kinds of pollutants such as radioactive, chemical and pharmaceutical wastes and pathogenic microorganisms (1). Wastewater discharge into the hospital surroundings can be an important factor to pathogens proliferation such as Enterobacteriaceae family like *Escherichia coli* strains in the environment (2, 3). Although *E. coli* bacteria in general, are not pathogenic, pathogenic strains of these bacteria have been observed. For example, urinary tract infection *E.coli* can be named (4– 6). Pollution of drinking or pool water with some pathotypes of *E. coli* has resulted in waterborne disease outbreaks and associated mortality (7, 8). If *E. coli* is found in the environment, can indicate the presence of other intestinal bacteria, because environmentally adapted strains of *E. coli* can stay a long time in the environmental water (9). There is differential survival and even growth of some *E. coli* strains from human feces in primary gut habitats compared to secondary habitats including soils, sediments, and water (10, 11).

There are other concerns of public health; one of them is the presence of antimicrobial resistant *E. coli* in the environment and a variety of resistance to antibiotics such as quinolones has been observed among them (12–14). Of note, plasmids or transposons can transfer antibiotic resistance genes between cells by transductive or conjugative processes. Spread of multidrug-resistant (MDR) bacteria is the result of it, in the further (15–18).

Quinolones, a family of widely used synthetic antimicrobial agents with a broad antibacterial spectrum, has very potent activity against Enterobacteriaceae including *E. coli*. As a result they have become common in human medicine (14, 19). Inhibiting the normal functions of bacterial type II topoisomerase is the target of quinolones (20). Resistance to quinolones mainly results from chromosomal point mutations in the quinolone resistance-determining regions (QRDRs) of DNA gyrase (*gyrA* and *gyrB*) and topoisomerase IV (*parC* and *parE*) encoding genes (21). Meanwhile, changes in the expression of efflux pumps and protection of DNA gyrase by the *qnr* protein, produced from plasmid-mediated quinolone resistance (PMQR) genes on a conjugative plasmid or transposon, have been demonstrated to cause quinolone resistance (9, 12). The presence of *qnr* genes may increase the selection of mutations with high-level quinolone resistance (22).

Considering the importance of hospital wastewaters as potential reservoirs for the dissemination of bacterial pathogens such as *E. coli* and antibiotic resistance genes in the environment, the need for such information becomes imperative (9). To the best of our knowledge, no studies have examined the prevalence of PMQR genes among the quinolone-resistant *E. coli* isolated from hospital wastewater sources in Tehran, Iran.

Therefore, we have investigated the frequency of quinolone resistance and PMQR genes (*qnrA*, *qnrB*, *qnrS*) in *E. coli* strains, isolated from different hospital wastewater sources in Tehran, since this species (i) is the most frequent enterobacterial species identified from human specimens and (ii) is involved in nosocomial and community-acquired infections.

## Materials and Methods

### Reagents and Media

Eosin methylene blue agar (EMB), trypticase soy broth (TSB), and Mueller-Hinton agar (MHA) were purchased from Merck (Darmstadt, Germany), while antibiotics were purchased from Oxoid (Basingstoke, UK). The other materials were purchased from Sigma (St. Louis, MO, USA).

### Water sample collection and E. coli isolation

The present study included 80 non-duplicate *E*. *coli* isolates originated from hospital wastewater sources over a 24-month sampling period (Jun 2014– Jun 2016). Sampling bottles were immediately placed in a lightproof insulated box containing icepacks to ensure rapid cooling. In order to further analysis, the samples were shipped to the Baqiyatallah Research Center Laboratory, Tehran, Iran. For culture-based tests, conventional methods were used according to Standard Methods for the Examination of Water and Wastewater 22*^nd^* edition. All samples were analyzed for the presence of *E*. *coli* (23). *E*. *coli* colonies from agar plates were picked and streaked for purity on EMB agar. Well-isolated colonies of purified *E. coli* were resuspended in trypticase soy broth with 20% (v/v) glycerol and stored in −70 °C for long-term storage, as described previously (24).

### Antibiotic susceptibility testing

All isolates were screened for quinolone- and fluoroquinolone- resistance phenotype using the Kirby-Bauer disk diffusion method according to the Clinical Laboratory Standards Institute guidelines (CLSI, 2013). The following antibiotics were used for antimicrobial susceptibility testing: ciprofloxacin (5 μg), nalidixic acid (30 μg), and norfloxacin (10 μg). The reference strain *E. coli* ATCC 25922 was used for quality control.

### DNA extraction and qnr genes amplification

The DNA of *E. coli* isolates found to be resistant to at least one antibiotic in the Kirby-Bauer disk diffusion method were extracted using a DNA extraction kit (Bioneer, Daejeon, South Korea) according to the manufacturer’s instructions, and was used for subsequent polymerase chain reaction (PCR) analysis.

PMQR determinants (*qnrA*, *qnrB*, and *qnrS*) from the quinolone-resistant strains were amplified using the primer sets listed in Table 1. Amplification was performed using a thermal cycler (Eppendorf, Hamburg, Germany) for 30 cycles. The PCR amplification reaction mixture consisted of 1 μl of each primer, 2.5 μl PCR buffer (10X), 0.7 μl MgCl*_2_* (50 mM), 0.7 μl of each 10 mM dNTP (MBI Fermentas, Vilnius, Lithuania), 0.5 μl of 5U Pfu DNA polymerase and 1 μl of sample DNA. PCR assays consisted of denaturation at 94 °C for 1 min, annealing of primers at 51–53 °C for 1 min, primer extension at 72 °C for 1 min, and the final extension at 72 °C for 7 min. To ascertain expected sizes of the amplicons, the PCR products were separated by electrophoresis at 100 V for 2 h on 1.5% (w/v) agarose gels, stained with ethidium bromide, and visualized using an ultra-violet (UV) transilluminator (Tanon, Shanghai, China). The positive controls for each gene were kindly provided by Dr. M. Memariani (Molecular Biology Research Center, Baqiyatallah University of Medical Sciences, Tehran, Iran).

**Table 1:** PCR primers used in the study for detection of *qnr* genes in *E. coli* isolates

**Primer/ target amplicon**	**Primer Sequence (5′→3′)**	**Amplicon size (bp)**	**Annealing temperature (°C)**	**Reference**
***qnrA***	F: ATT TCT CAC GCC AGG ATT TG R: GAT CGG CAA AGG TTA GGT CA	516	53	(18)
***qnrB***	F: GTT GGC GAA AAA ATT GAC AGA A R: ACT CCG AAT TGG TCA GAT CG	526	53	(25)
***qnrS***	F: ACG ACA TTC GTC AAC TGC AA R: TTA ATT GGC ACC CTG TAG GC	417	51	(18)

## Results

In total, 80 *E. coli* isolates were isolated during the study period, of which 51 (63.8%) isolates were resistant to tested antibiotics (Table 2).

**Table 2:** Prevalence of quinolone resistance and their combinations among *E. coli* isolates

**Antibiotic resistance**	**n (%)**
**One antibiotic**	
CIP	2 (3.9)
NAL	21 (41.2)
NFX	0 (0)
Total	23 (45.1)
**More than one antibiotic**	
NFX + CIP	1 (2)
NFX + NAL	10 (19.6)
CIP + NAL	4 (7.8)
NFX + CIP +NAL	13 (25.5)
Total	28 (54.9)

Abbreviation: CIP, ciprofloxacin; NAL, nalidixic acid; NFX, norfloxacin

Among antibiotic-resistant isolates, 45.1% were resistant to one antibiotic and 54.9% were resistant to more than one antibiotic (Table 2). Of note, 13 (25.5%) of isolates were resistant to all the antibiotics employed. Among 80 isolates, the highest rates of antibiotic resistance were obtained for nalidixic acid (60%), followed by norfloxacin (30%), and ciprofloxacin (25%).

**Fig. 1:**
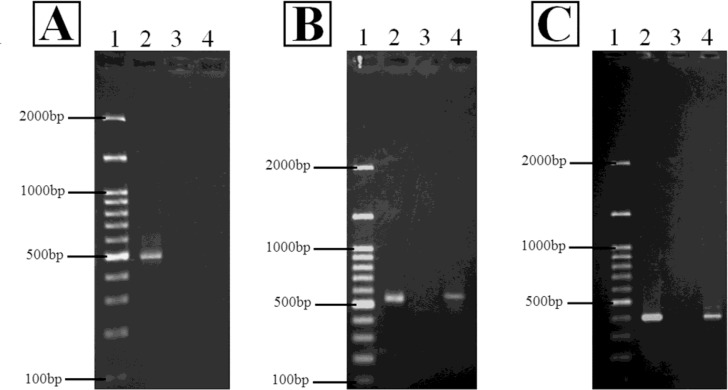
PCR amplification of the *qnr* genes using the primers *qnrA* (A) and *qnrB* (B), and *qnrS* (C), Lanes 1, 2, 3, and 4 represent DNA ladder, positive control, negative control, and *qnr* gene, respectively.

The results of DNA amplification by the PCR method based on the primers described in Table 1 showed the presence of 526-bp and 417-bp fragments for the PMQR genes amplified by *qnrB* and *qnrS* primers, respectively (Fig. 1). None of the isolates harboured the *qnrA* gene (Table 3).

**Table 3:** Prevalence of *qnr* genes and their combinations among quinolone resistance isolates

**Genes**	**n (%)**
**One gene**	
*qnrA*	0 (0)
*qnrB*	11 (45.8%)
*qnrS*	7 (29.2%)
Total	18 (75%)
**More than one gene**	
*qnrA + qnrB*	0 (0)
*qnrA + qnrS*	0 (0)
*qnrB + qnrS*	6 (25%)
*qnrA + qnrB + qnrS*	0 (0)
Total	6 (25%)

Of the 51 quinolone-resistant strains, 24 (47.1%) isolates harboured *qnr* genes. According to the PCR results, 11 (45.8%) and 7 (29.2%) isolates contained *qnrB* and *qnrS*, respectively. In addition, 6 (25%) isolates harboured both *qnrB* and *qnrS* genes (Table 3). Among these 6 strains, 5 isolates were resistant to all the antibiotics tested. The most common PMQR gene was *qnrB*, observed in 17 isolates.

## Discussion

High levels of antibiotics are typically discharged in human and hospital wastes, sufficient to inhibit susceptible bacteria and provide a selective advantage to resistant bacteria (26). The population of resistant bacteria and antibiotic resistance gene transfer to human pathogenic bacteria can be done easier in the aquatic environment. It increases the problem of the development of clinical drug resistance (27).

There are various sources of antibiotic resistance. Hospital wastewater has a special role among them; this is because it is dissemination. When the wastewater that containing resistance elements moves, the spread of antimicrobial resistance can also is performed (28). Inside the hospital waste, drug-resistant bacteria and active antimicrobials are seen together (29).

In the present work, we have observed that *E. coli* recovered from the hospital wastewaters showed relatively high rates of quinolone resistance (63.8%), wastewater of hospitals is an important source of quinolone resistance in Tehran, Iran. The relatively high level of resistance to quinolones recorded in the present study could be attributed to misuse or abuse of these antibiotics. Based on the results of the present study, the highest rate of antibiotic resistance was obtained for nalidixic acid (60%). This finding was higher than that expressed in previous reports from Hamadan, Iran (30). Previous study from India revealed that the resistance to cephalosporins and quinolones was more frequent than to aminoglycosides and imipenem in *E. coli* isolated from hospital wastewaters (31). In the other study conducted in Leon, Nicaragua, many *E. coli* strains isolated from waste and other water sources. Those isolated from the hospital wastewater had higher antibiotics resistance compared with others (32). They reported a resistance rate of 70% and 69% for nalidixic acid and ciprofloxacin, respectively, which was higher than that of our findings. Quinolones are used in medicine and their stability in water is very high (33). Moreover, if someone, be exposed to quinolones in a long time, the likelihood of developing resistance to these antibiotics will increase (34). Therefore, probably, it is the source of an important driving force for the selection of resistance to quinolone (33).

According to our results, among the 51 quino-lone-resistant *E. coli* isolates, *qnr* genes were present in 24 (47.1%) isolates, which was higher than that of previous study conducted in India (31). The results of this study regarding *qnrA* gene are in accordance with recent reports, that showed none of the isolates, recovered from hospital wastewater, were positive for *qnrA* gene (28, 35, 36). In this study, *qnrB* was the most frequent genes in *E. coli* isolates. This trend is in agreement with previous surveillance (37), *qnrB* was the most common *qnr* genes among *E. coli* isolated from hospital wastewater from central India.

PMQR determinants were discovered in 1998. From that time until now, they are reported as a multifaceted threat on worldwide (31). The outbreak of these factors largely depends on the selection criteria of studied strains; change from 16.7% to up to 58.0% (38). The high rate of PMQR determinants among *E. coli* isolates is a serious public health concern since these isolates have the potential to spread their resistance genes to other environmental bacteria. Noticeably, the PMQR determinants are located on mobile genetic elements, which may allow for dissemination among members of the Enterobacteriaceae family (18). Therefore, long-term surveillance is required for control the further spread of these threatening agents.

## Conclusion

This study constitutes the first epidemiological survey of the quinolone resistance and PMQR determinants among *E. coli* strains isolated from hospital wastewater sources in Tehran, Iran, and identified the *qnrB* was the most common PMQR gene. Our findings showed high rates of quino-lone resistance (63.8%) and PMQR genes, underlining the importance of hospital wastewaters as reservoirs for dissemination of potentially pathogenic *E. coli* and horizontal gene transfer between other waterborne bacterial species. To gain more insight into the molecular characterization of quinolone-resistant *E. coli* isolates, other possible mechanisms of resistance such as QRDR, changes in expression of efflux pumps, or even novel mechanisms should also be investigated.

## Ethical considerations

Ethical issues (Including plagiarism, informed consent, misconduct, data fabrication and/or falsification, double publication and/or submission, redundancy, etc.) have been completely observed by the authors.
